# Efficacy of natural killer cell activity as a biomarker for predicting immunotherapy response in non‐small cell lung cancer

**DOI:** 10.1111/1759-7714.13677

**Published:** 2020-10-05

**Authors:** Myeong Geun Choi, Yeon Joo Kim, Jae Cheol Lee, Jin Kyung Rho, Chang‐Min Choi

**Affiliations:** ^1^ Department of Pulmonary and Critical Care Medicine, Asan Medical Center University of Ulsan College of Medicine Seoul South Korea; ^2^ Department of Oncology, Asan Medical Center University of Ulsan College of Medicine Seoul South Korea; ^3^ Department of Convergence Medicine, Asan Medical Center University of Ulsan College of Medicine Seoul South Korea

**Keywords:** Biomarker, immunotherapy, natural killer cell, non‐small cell lung cancer

## Abstract

**Background:**

The establishment of biomarkers that can be used to predict the response to immunotherapy for malignancy is extremely important. In particular, noninvasive analysis of immune cells from peripheral blood before treatment has gained increased attention, and natural killer (NK) cell activity has been shown to be related to treatment response. Here, we aimed to confirm the relationship between the response to immunotherapy and NK cell activity.

**Methods:**

In this prospective observational study, patients with advanced NSCLC who were scheduled for immunotherapy from October 2018 to December 2019 were enrolled. Baseline NK cell activity was compared according to the best clinical response to immunotherapy.

**Results:**

A total of 54 patients with advanced NSCLC were enrolled, and 34 patients were analyzed. The baseline NK cell activity was significantly higher in the non‐PD group than in the PD group (*P* = 0.002). At the cutoff level of ≥1200 pg/mL, baseline NK cell activity yielded a sensitivity of 80% and a specificity of 68.4% in predicting the response to immunotherapy (AUC = 0.8, *P* < 0.003). The median progression‐free survival (PFS) was significantly better in the high NK group (*P* = 0.003), and correlation between baseline NK cell activity and PFS was also confirmed (r = 0.517, *P* = 0.002).

**Conclusions:**

Baseline NK cell activity was related to the response to immunotherapy and the PFS. We suggest that NK cell activity from peripheral blood before immunotherapy is a noninvasive, simple, and novel way to predicting the treatment response in patients with NSCLC.

**Key points:**

**Significant findings of the study:**

- The response to immunotherapy was significantly better in patients with high baseline NK cell activity, and there was a significant correlation between baseline NK cell activity and PFS.

**What this study adds**

- This study demonstrates the efficacy of baseline NK cell activity from peripheral blood as a biomarker for predicting immunotherapy response.

## Graphical abstract

In this prospective observational study, patients with advanced NSCLC who were scheduled for immunotherapy from October 2018 to December 2019 were enrolled. The baseline NK cell activity was significantly higher in the non‐PD group than in the PD group. NK cell activity from peripheral blood before immunotherapy is a non‐invasive, simple, and novel way to predicting the treatment response in patients with NSCLC.

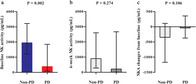

## Introduction

The efficacy of cancer immunotherapy has been confirmed in recent years in several malignant diseases, including non‐small cell lung cancer (NSCLC), and has become an innovative treatment modality in patients with malignancy.^1^, ^2^, ^3^ In particular, checkpoint inhibitors targeting programmed cell death‐1 (PD‐1)/programmed cell death‐ligand 1 (PD‐L1), have received considerable interest, and their clinical utility is developing rapidly. PD‐L1 is usually found in tumor cells, whereas PD‐1 is found in immune cells, such as CD4 T cells, CD8 T cells, B cells, monocytes, dendritic cells, and NK cells.^4^ The use of PD‐1/PD‐L1 antibodies block these ligand‐receptor interactions, while augmenting T cell proliferation, and consequently suppressing the antitumor response.^5^ In other words, by suppressing these checkpoint inhibitors, this negative regulation can be reversed and subsequently lead to reactivation of the antitumor immune response.

However, despite this concept, clinically, the response rates of checkpoint inhibitors are only about 20% and treatment often fails.^1^, ^2^, ^3^ Although the reasons for this lack of efficacy are somewhat unknown, it is thought to result from the complexity of the immune checkpoint pathway, as well as the diverse array of tumor escape mechanisms.^6^ Numerous studies have sought to predict the response to immunotherapy by measuring biomarkers such as PD‐L1 expression, tumor mutational load, and T cell repertoire sequencing^7^, ^8^, ^9^; however, recent studies have shown contrasting results.^10^ Furthermore, these biomarkers require invasive biopsy, have a heterogenous result according to biopsy site, and often carry a high cost and adverse events. Therefore, biomarkers that can be obtained noninvasively to predict the response to immunotherapy are urgently required.

As the host immune system is known to play an important role in the response to immunotherapy,^11^ the association between the response to immunotherapy and the characteristics of the immune cells from peripheral bloods, including subset composition, function, cytokines, and proteins has been revealed.^12^ Usually, the baseline or on treatment fraction of the effector T cells are associated with a good outcome for immunotherapy, while a high fraction of inhibitory T cells is related to poor outcome.^13^ PD‐1/PD‐L1 inhibitor kills tumor cells, which express human leukocyte antigen‐1 (HLA‐1), by increasing T cell activity; however, HLA‐1 negative tumor cells can escape from immunotherapy through “missing self”.^14^ Tumor cells that avoid T cell mediated killing can be recognized and killed by natural killer (NK) cells, an innate lymphocyte population with cytotoxic activity.^15^ Recent studies have examined PD‐1 expression on NK cells, and it is known that the response to PD‐1/PD‐L1 inhibitors is related to NK cells.^16^

In our previous study, the frequency and function of immune cells, including NK cells, in a total of nine patients were evaluated in order to determine biomarkers related to the early response to immunotherapy.^17^ To this end, we found that the baseline NK cell activity was significantly higher in the responders. The purpose of the current study was to confirm the relationship between the response to immunotherapy and NK cell activity, and to identify additional biomarkers related to the response to immunotherapy.

## Methods

### Study design and patients

This was a prospective cohort observational study conducted on patients with advanced NSCLC scheduled for immunotherapy from October 2018 to December 2019. All patients were pathologically diagnosed with NSCLC, and adult patients between the ages of 20 and 80 were enrolled. The exclusion criteria were those with an Eastern Cooperative Oncology Group (ECOG) performance score >2, and those accompanied by malignancy other than NSCLC within five years of the NSCLC diagnosis. Peripheral blood was collected from patients before and six weeks after the immunotherapy. The number of subjects in this preliminary study was calculated based on the study period and the number of patients who received immunotherapy prior to this study. All participants provided their written informed consent, and the study was approved by the Institutional Review Board of the Asan Medical Center (IRB No. 2018–0755).

### Clinical response assessment and outcomes

The primary study outcome was the comparison of baseline NK cell activity according to the clinical response to immunotherapy. The response was evaluated using computed tomography (CT) scan and immune‐related response criteria (irRC).^18^ Patients were included in the non‐PD group if their best response was partial response (PR) or stable disease (SD), while patients were included in the PD group if the best response was progressive disease (PD). Unless the patient was unstable, the assessment of response was performed at least four weeks after the initiation of immunotherapy and re‐evaluated every two or three cycles. If cancer progression was suspected by CT scan, pseudo‐progression was ruled out with a consecutive CT scan.

The secondary endpoints were NK cell activity at six weeks, and the change in NK cell activity from baseline. The cutoff baseline NK cell activity and its sensitivity and specificity were evaluated for the best prediction of disease control with immunotherapy. The baseline characteristics that could affect the progression‐free survival (PFS), such as PD‐L1 status, tumor mutational burden, epidermal growth factor receptor (*EGFR*) mutation, and complete blood count (CBC) profile, were also included as secondary outcomes.

### NK cell activity

The NK cell activity was measured using the NK Vue kit (ATgen, Sungnam, Korea) according to the manufacturer's instructions. When NK cells were exposed to a specific recombinant cytokine, Promoca (ATgen), the release of IFN‐γ was measured by quantitative sandwich enzyme‐linked immunosorbent assay (ELISA). The concentration of IFN‐γ can be used as a surrogate marker to measure NK cell activity in peripheral blood. Briefly, 1 mL of whole blood was collected into a presupplied Promoca tube and gently inverted three times. The tube was then incubated for 24 hours in a chamber at a temperature of 37°C. A CODA microplate processor (Biorad, Hercules, CA) was used to measure the optical density (OD), and standard curves were created using seven standards in duplicate. The OD values were then entered into NK Vue software, and the results were calculated in pg/mL.

### Statistical analysis

The data obtained from patients were used to analyze the baseline and demographic characteristics. Mann‐Whitney U test was used to compare continuous variables, and Fisher's exact test was used for categorical variables. Receiver operating characteristic (ROC) curve analysis was used to establish the cutoff value of NK cell activity to predict disease control or progression, as the best response to immunotherapy. Spearman correlation was used to analyze the relationship between PFS and baseline NK cell activity, and Kaplan‐Meier estimation and Log‐rank test were used for PFS and survival analysis. Univariate and multivariate hazard ratios were calculated using Cox regression analysis to investigate the baseline characteristics affecting PFS. A *P*‐value < 0.05 was considered statistically significant. Statistical analyses were performed using IBM SPSS version 25.0 (IBM Corp., Armonk, NY, USA).

## Results

### Patient baseline characteristics

**Figure 1:**
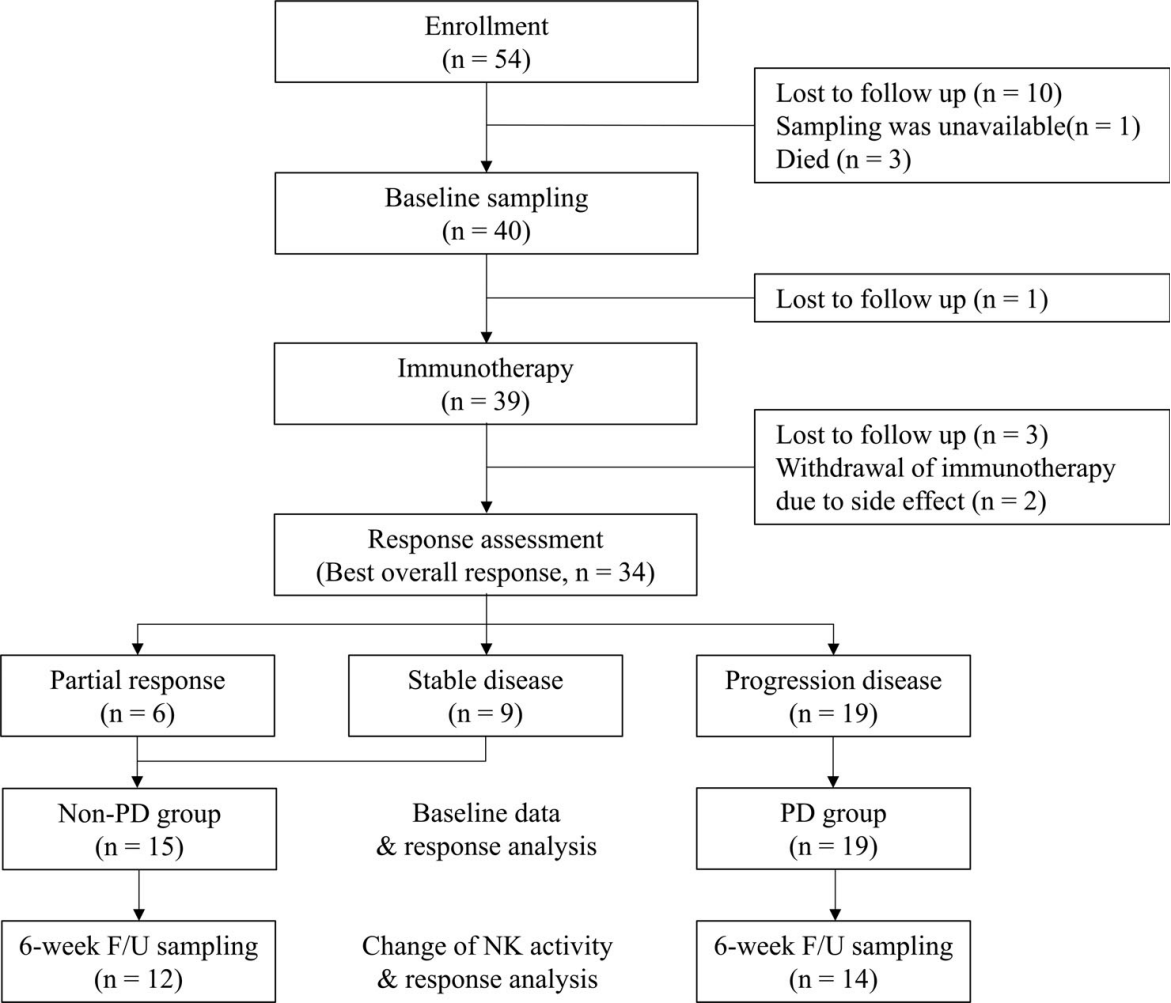
Consort flow diagram.

From October 2018 to December 2019, a total of 54 patients with advanced NSCLC who met the inclusion and exclusion criteria were enrolled. Among them, baseline blood sampling was performed on 40 patients, and 34 patients received at least one response assessment after immunotherapy. Of these patients, six had PR as their best response, nine had SD, and 19 had PD. In the non‐PD group and PD group, 12 and 14 patients, respectively, six‐week follow‐up blood sampling was conducted, and NK cell activity changes from baseline were analyzed (Fig 1).

The baseline characteristics of the non‐PD group and PD group were similar (Table 1). The median age of patients from the non‐PD group and PD group was 69 and 59 years old, respectively, but there was no significant difference. In both groups, the proportion of males was higher than that of females, and more than half of the patients were diagnosed with adenocarcinoma. Most patients had an ECOG performance score of 0 or 1, and the majority of patients had a smoking history. All patients received standalone immunotherapy as second‐ or later‐line of treatment. Previous treatments were targeted therapy or standard chemotherapy, and immunotherapy was administered after the blood cells fully recovered from the previous treatment. The proportion of immunotherapy drugs administered to patients was similar among pembrolizumab, nivolumab, and atezolizumab. Approximately 20% of patients had an *EGFR* mutation, half of patients had wild‐type, and approximately 30% of patients, including patients with squamous cell carcinoma, were not identified with EGFR status. PD‐L1 expression was positive in most patients, and the distribution of patients according to expression frequency was similar between both groups.

**Table 1:** Baseline characteristics

	Total (*n* = 34)	Non‐PD group (*n* = 15)	PD group (*n* = 19)	*P*‐value
Age, years (median)	64	69	59	0.096†
Sex, *n* (%)				1.000‡
M	26 (76.5)	12 (80)	14 (73.7)	
F	8 (23.5)	3 (20)	5 (26.3)	
Morphology, *n* (%)				0.717‡
Adenocarcinoma	20 (58.8)	8 (53.3)	12 (63.2)	
Squamous cell carcinoma	13 (38.2)	6 (40)	7 (36.8)	
Others	1 (2.9)	1 (6.7)	0 (0)	
ECOG score				1.000‡
0–1	29 (85.3)	13 (86.7)	16 (84.2)	
2	5 (14.7)	2 (13.3)	3 (15.8)	
Smoking, *n* (%)				0.715‡
Never	11 (32.4)	4 (26.7)	7 (36.8)	
Former	23 (67.6)	11 (73.3)	12 (63.2)	
Lines of prior therapy, *n* (%)				0.920‡
1	21 (61.8)	10 (66.7)	11 (57.9)	
2	6 (17.6)	2 (13.3)	4 (21.1)	
≥3	7 (20.6)	3 (20)	4 (21.1)	
Immunotherapy, *n* (%)				0.355‡
Pembrolizumab	12 (35.3)	4 (26.7)	8 (42.1)	
Nivolumab	7 (20.6)	5 (33.3)	2 (10.5)	
Atezolizumab	15 (44.1)	6 (40)	9 (47.4)	
*EGFR* mutation, *n* (%)				NA
Mutation	7 (20.6)	3 (20)	4 (21.1)	
Wild‐type	16 (47.1)	7 (46.7)	9 (47.4)	
Not done	11 (32.4)	5 (33.3)	6 (31.6)	
PD‐L1 expression, *n* (%)				NA
22C3				
≥50%	8 (23.5)	2 (13.3)	6 (31.6)	
1%–49%	9 (26.5)	7 (46.7)	2 (10.5)	
<1%	8 (23.5)	3 (20)	5 (26.3)	
Unknown	9 (26.5)	3 (20)	6 (31.6)	
SP263				
≥50%	15 (44.1)	6 (40)	9 (47.4)	
1%–49%	8 (23.5)	5 (33.3)	3 (15.8)	
<1%	10 (29.4)	3 (20)	7 (36.8)	
Unknown	1 (2.9)	1 (6.7)	0 (0)	
Median PFS, days (IQR)	65 (37, 146)	185 (78, N/E)	38 (34, 49)	
Median OS, days (IQR)	246 (105, N/E)	N/E (246, N/E)	146 (61, 438)	

† Mann–Whitney U test.

‡ Fisher's exact test.

ECOG, Eastern Cooperative Oncology Group; EGFR, epidermal growth factor receptor; N/E, not estimable; OS, overall survival; PD‐L1, programmed cell death‐ligand 1; PFS, progression‐free survival.

### NK cell activity as a biomarker for predicting response to immunotherapy

**Figure 2:**
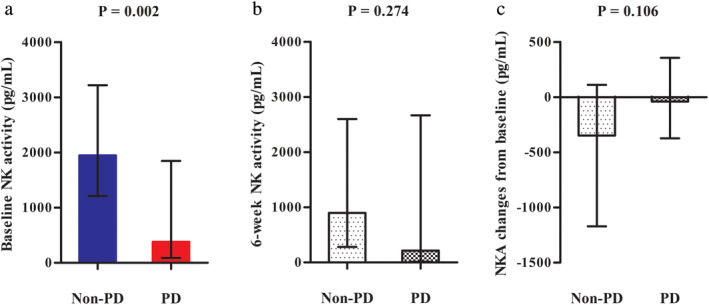
(**a**) Baseline natural killer (NK) cell activity. (**b**) Six‐week NK cell activity after initiation of immunotherapy. (**c**) NK cell activity changes from baseline. Error bars represent interquartile range of median.

**Figure 3:**
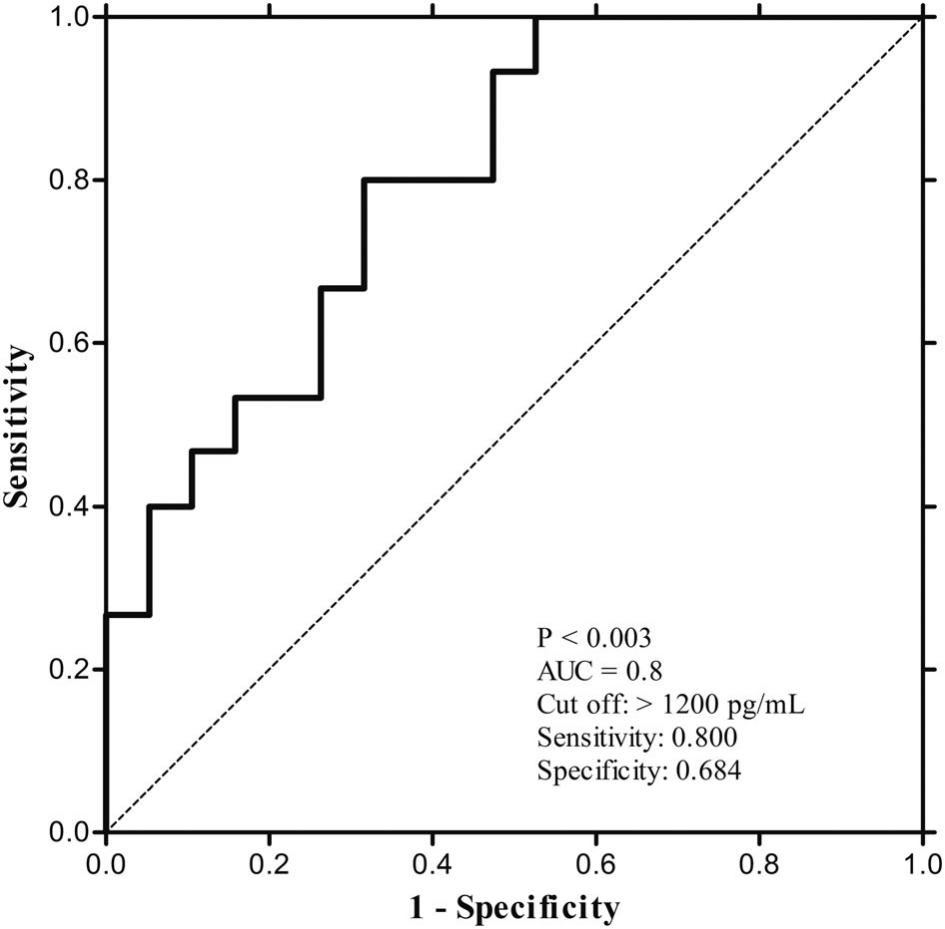
ROC curve of NK cell activity for predicting disease control (partial response and stable disease) in patients who received immunotherapy. (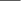) Baseline NKA (pg/mL), (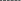) Reference line

The baseline NK cell activity was significantly higher in the non‐PD group than the PD group. (1946.00 pg/mL vs. 382.80 pg/mL, respectively; *P* = 0.002; Fig 2a) Follow‐up NK cell activity six weeks after immunotherapy was also higher in the non‐PD group than the PD group, but there was no statistical significance (898.25 pg/mL vs. 213.20 pg/mL, respectively; *P* = 0.274; Fig 2b) Both groups showed a decrease in NK cell activity six weeks from baseline, but there was no significant difference change in NK cell activity (−347.65 pg/mL vs. −41.10 pg/mL, respectively; *P* = 0.106; Fig 2c). The ROC curve for baseline NK cell activity used to predict the response to immunotherapy is shown in Fig 3. At a cutoff level of ≥1200 pg/mL, the baseline NK cell activity yielded a sensitivity of 80% and a specificity of 68.4% in predicting controlled disease with immunotherapy (AUC = 0.8, *P* < 0.003). Therefore, baseline NK cell activity was considered to be a meaningful predictive tool.

### Baseline NK cell activity and survival

**Figure 4:**
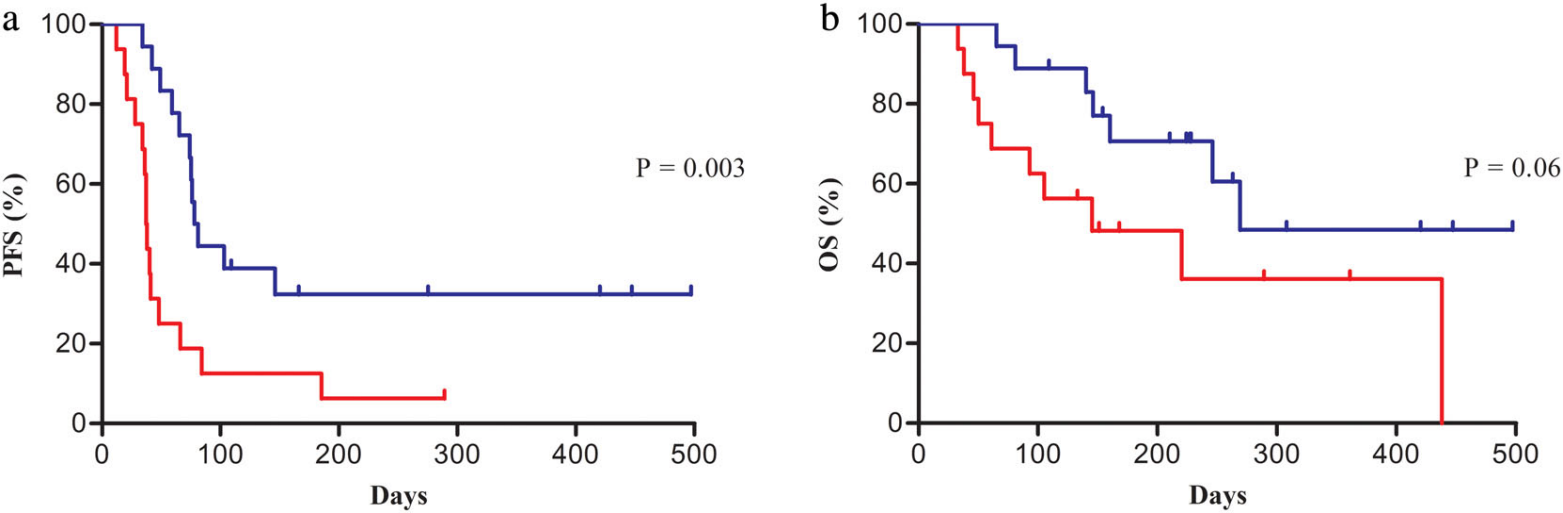
Kaplan‐Meier curves according to baseline NK cell activity (cutoff value: 1200 pg/mL). (**a**) Progression‐free survival. (**b**) Overall survival. (**a**) (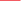) NKA <1200, (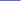) NKA ≥1200; (**b**) (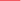) NKA <1200, (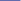) NKA ≥1200

The median follow‐up period of all study participants was 246 days. The median PFS was 65 days in the total patients, 185 days in the non‐PD group, and 38 days in the PD group, and 17 of 34 patients died during the follow‐up period. The median overall survival (OS) was 246 days for all enrolled patients. Survival analysis was conducted in two groups; the high NK group, and the low NK group, divided by baseline NK cell activity based on 1200 pg/mg (Table 2 and Fig 4). The median PFS was significantly longer in the high NK group (78 days) compared to that of the low NK group (37 days) (*P* = 0.003). With regards to OS, the median OS was 269 days in the high NK group and 145 days in the low NK group, but there was no statistical significance (*P* = 0.06). A significant correlation was shown between baseline NK cell activity and PFS, in which the PFS was longer and the baseline NK cell activity increased (r = 0.517, *P* = 0.002; Table 2). In contrast, no correlation was found between baseline NK cell activity and OS.

**Table 2:** Comparison of progression‐free survival (PFS) and overall survival (OS) according to baseline NK cell activity (pg/mL) and Spearman correlation coefficients for the relationship between baseline NK cell activity and PFS and OS

					Baseline NK activity	
	NKA ≥1200 (*n* = 18)	NKA <1200 (*n* = 16)	Total (*n* = 34)	*P*‐value^†^	Correlation coefficient (r)	*P*‐value^‡^
Progression‐free survival (PFS)				0.003	0.517	0.002
Patients with event, *n* (%)	12 (66.7)	15 (93.8)	27 (79.4)			
Median PFS, days (IQR)	78 (65, N/E)	37 (28, 48)	65 (37, 146)			
Mean PFS, days (95% CI)	211 (118, 305)	63 (29, 98)	146 (85, 206)			
Overall survival (OS)				0.06	0.279	0.109
Patients with event, *n* (%)	7 (38.9)	10 (62.5)	17 (50)			
Median OS, days (IQR)	269 (160, N/E)	145 (50, 438)	246 (105, N/E)			
Mean OS, days (95% CI)	333 (243, 423)	223 (128, 319)	285 (218, 353)			

^†^ Log‐rank test.

^‡^ Spearman correlation.

95% CI, 95% confidence interval; IQR, interquartile range; NKA, natural killer cell activity.

### PD‐L1 status and response to immunotherapy

PFS and OS according to PD‐L1 status were also analyzed (Table S1 and Fig S1). Of the total patients, 24 were positive for PD‐L1 and 10 were negative. The median PFS in PD‐L1 positive patients was 66 days, and the median PFS in PD‐L1 negative patients was 49 days. The OS of PD‐L1 positive and negative patients was 269 and 246 days, respectively. The PFS and OS in PD‐L1 positive patients were slightly longer than those observed in PD‐L1 negative patients, but there was no statistical significance.

### Univariate and multivariate analyses

Univariate and multivariate analyses were conducted on whether baseline characteristics, PD‐L1 status, CBC profile, or NK cell activity affected the PFS (Table 3). The hazard ratio of patients with an ECOG PS score ≥2 was 1.428, whereas the hazard ratio was <1, with high tumor mutational burden or positivity for PD‐L1, but no significant difference was observed. With regards to the CBC profile, the hazard ratio for a high neutrophil‐lymphocyte ratio was 2.514, but no statistical significance was observed in multivariate analysis. If the baseline NK cell activity was ≥1200 pg/mL, the hazard ratio in multivariate analysis was 0.377 (*P* = 0.016).

**Table 3:** Univariate and multivariate hazard ratios by Cox regression analysis for progression‐free survival

	Progression‐free survival	
Variables	HR (95% CI)	*P*‐value
Univariate analysis		
Baseline characteristics		
Age ≥60	0.617 (0.286, 1.333)	0.219
Sex, male	0.784 (0.327, 1.881)	0.586
Morphology, adenocarcinoma	1.022 (0.467, 2.237)	0.957
Ever smoker	0.722 (0.329, 1.586)	0.418
ECOG score ≥2	1.428 (0.537, 3.796)	0.475
Lines of prior therapy ≥2	1.106 (0.505, 2.424)	0.802
EGFR, mutation	1.035 (0.367, 2.922)	0.948
Tumor mutational burden >10 mut/Mb	0.610 (0.161, 2.314)	0.468
PD‐L1 status		
PD‐L1 ≥1%	0.721 (0.322, 1.615)	0.426
PD‐L1 ≥5%	0.678 (0.314, 1.467)	0.324
PD‐L1 ≥50%	0.993 (0.464, 2.126)	0.985
CBC profile		
WBC >10 000	0.857 (0.295, 2.486)	0.776
ANC >7000	0.647 (0.194, 2.156)	0.478
ALC <900	1.518 (0.565, 4.081)	0.408
AMC >630	1.151 (0.540, 2.456)	0.716
ANC/ALC ≥3.0	2.514 (1.159, 5.453)	0.020
M/L >11.3	0.987 (0.132, 7.370)	0.990
Platelet >450 000	0.548 (0.164, 1.834)	0.329
NK activity		
Baseline NK activity ≥1200 pg/mL	0.325 (0.150, 0.705)	0.004
Multivariate analysis		
ANC/ALC ≥3.0	2.051 (0.918, 4.581)	0.080
Baseline NK activity ≥1200 pg/mL	0.377 (0.170, 0.835)	0.016

ALC, absolute lymphocyte count; AMC, absolute monocyte count; ANC, absolute neutrophil count; CBC, complete blood count; HR, hazard ratio; M/L, monocyte/lymphocyte; WBC, white blood cell.

## Discussion

This study demonstrates the efficacy of baseline NK cell activity as a biomarker for predicting response to immunotherapy, in particular PD‐1/PD‐L1 inhibitors. The response to immunotherapy was significantly better in patients with high baseline NK cell activity, and there was significant correlation between baseline NK cell activity and PFS. We established the cutoff baseline NK cell activity as ≥1200 pg/mL, and showed that this could predict the response to immunotherapy using ROC curve analysis. Furthermore, we were able to confirm that the PFS was improved in the high NK group, whose baseline NK cell was higher than this cutoff value, compared to the low NK group. Therefore, we suggest that physicians can easily and noninvasively predict response to immunotherapy, and make decisions on treatment regimens by obtaining peripheral blood from patients and measuring NK cell activity before treatment.

Traditionally, measuring biomarkers, such as PD‐L1 expression and tumor mutational burden, to predict the response to immunotherapy have required invasive biopsy.^11^, ^19^ In order to reduce this disadvantage, recent efforts have focused on establishing biomarkers from peripheral blood.^20^ Indeed, a previous study analyzed the outcome of immunotherapy according to immune cell count or frequency, including neutrophils, lymphocytes, and the neutrophil‐lymphocyte ratio from peripheral blood.^21^ In patients with melanoma, the biomarker predicting response to PD‐1/PD‐L1 inhibitors was studied using flow cytometry analysis, and it was shown that NK cell subsets were associated with the response to immunotherapy.^22^ However, no previous study has examined the relationship between NK cell function in peripheral blood and the response to immunotherapy in patients with NSCLC. Therefore, our study was meaningful and pioneering in that it confirmed the possibility of a relationship between NK cells and response to immunotherapy. In addition, to prove the result by using response as categorical outcome, we utilized PFS as a continuous outcome. A cutoff value of NK cell activity to predict the response was also suggested.

So far, the mechanism underlining the relationship between NK cell activity and PD‐1/PD‐L1 inhibitors has not been revealed, although there are some hypotheses.^15^, ^23^ In a mouse model, a PD‐1/PD‐L1 inhibitor augmented NK cell function, and NK cells with PD‐1 expression were found to be the main responders to PD‐1/PD‐L1 inhibitors in patients with HLA‐1 deficient tumors.^16^ Furthermore, it was shown that the greater the number of NK cells with PD‐1 expression, the better response to the PD‐1/PD‐L1 inhibitor. In contrast, response to immunotherapy was worse in the NK‐depleted group. Another hypothesis is that IFN‐γ secreted by NK cells may increase the response to PD‐1/PD‐L1 inhibitors as a result of increased expression of PD‐L1 in tumor cell.^24^, ^25^ The results of this study can also be explained by these hypotheses, although further study is required to fully elucidate the mechanisms.

In addition to findings related to NK cell activity, we also demonstrated that patients with a neutrophil‐lymphocyte ratio ≥3.0 were remarkably close to significance, with a hazard ratio of 2.5 in multivariate analysis. This result, similar to those of previous studies, supports that cytotoxic CD8 T cells are associated with a positive outcome, while neutrophil count is associated with a poor outcome.^21^, ^26^, ^27^ This suggests that a CBC profile conducted routinely before treatment may prove to be a useful tool to predict treatment response.

There are some limitations of this study. The first limitation was its small sample size; a total of 34 patients were finally analyzed in this preliminary study, and the results will need to be confirmed by large‐scale studies in the future. Another limitation was the short‐term study period. Although the correlation between NK cell activity and the best response or PFS was verified, the follow‐up period was too short to demonstrate the relationship with OS. As such, additional studies with longer follow‐up are required in the future. Moreover, the role of PD‐L1 expression, which is already known as a predictive biomarker, was not demonstrated in this study, possibly due to the abovementioned limitations.

Furthermore, it would be better if the response to immunotherapy was further subdivided. For example, if the hyperprogression, which has recently been in the spotlight,^28^ were to be analyzed separately in the PD group, clinically meaningful results could be obtained. Although data were not shown, baseline NK cell activity from three patients, who initiated immunotherapy but were lost to follow‐up before the response assessment, were measured low, with a mean of 78.13 pg/mL and a median of 53.6 pg/mL. If these patients were lost to follow‐up because of poor outcome, such as hyperprogression, there would be a possibility that NK cell activity and hyperprogression are also related. In addition, a further limitation may exist in that the blood samples were only collected at two points. Therefore, further study with more follow‐up blood sampling could reveal the association with change of measured values and prognosis.

PFS for all patients in this study was 65 days and shorter than the known PFS of immunotherapy.^1^, ^2^, ^3^ This is believed to be because the lines of treatment of these patients were second or later and had received various kinds of immunotherapy. In fact, the PFS for immunotherapy was 1.9–4.0 months in patients on second or above lines of treatment, and the PFS for nivolumab and atezolizumab was shorter than that for pembrolizumab.^29^, ^30^, ^31^, ^32^ The proportion of treatment lines or kinds of immunotherapy in the two groups did not statistically differ in this study; however, these variables should be controlled in further studies. Finally, patients only received a PD‐1/PD‐L1 inhibitor, and it is necessary to perform further studies with immunotherapies other than PD‐1/PD‐L1 inhibitors.

In conclusion, baseline NK cell activity from patients with NSCLC was related to the response and PFS to immunotherapy. Therefore, we suggest that NK cell activity from peripheral blood before immunotherapy is a noninvasive, simple, and novel laboratory test for predicting response of treatment in patients with NSCLC. Future long‐term and large‐scale studies with various immunotherapies are required to confirm the efficacy of NK cell activity as a predictive biomarker for prognosis.

## Disclosure

The authors have no conflicts of interest to declare.

## Supporting information


**Figure S1** Kaplan–Meier curves according to PD‐L1 expression. (**a**) Progression‐free survival. (**b**) Overall survival.
**Table S1** Progression‐free and overall survival according to PD‐L1 expression.
