# Supplementary material for: Efficacy of natural killer cell activity as a biomarker for predicting immunotherapy response in non‐small cell lung cancer
Source: Thorac Cancer. 2020 Oct 5;11(11):3337–45. doi: 10.1111/1759-7714.13677 (PMC7606014; doi:10.1111/1759-7714.13677)
Supplement: Supplementary file 1 — Figure S1 Kaplan–Meier curves according to PD‐L1 expression. (a) Progression‐free survival. (b) Overall survival. Table S1 Progression‐free and overall survival according to PD‐L1 expression. [file TCA-11-3337-s001.docx]

**Supplementary Figure 1.** Kaplan–Meier curves according to PD-L1 expression. (A) Progression-free survival. (B) Overall survival.


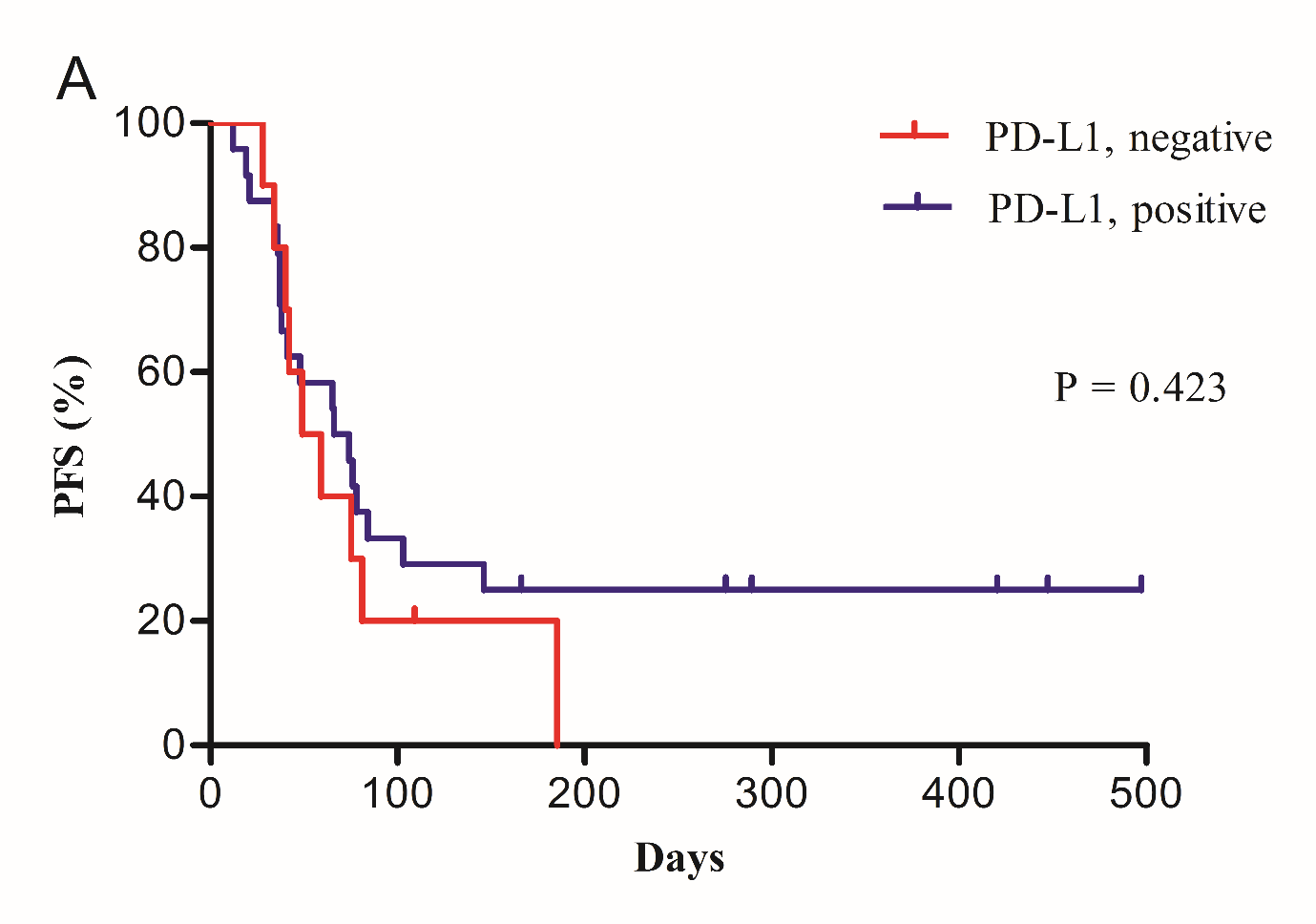

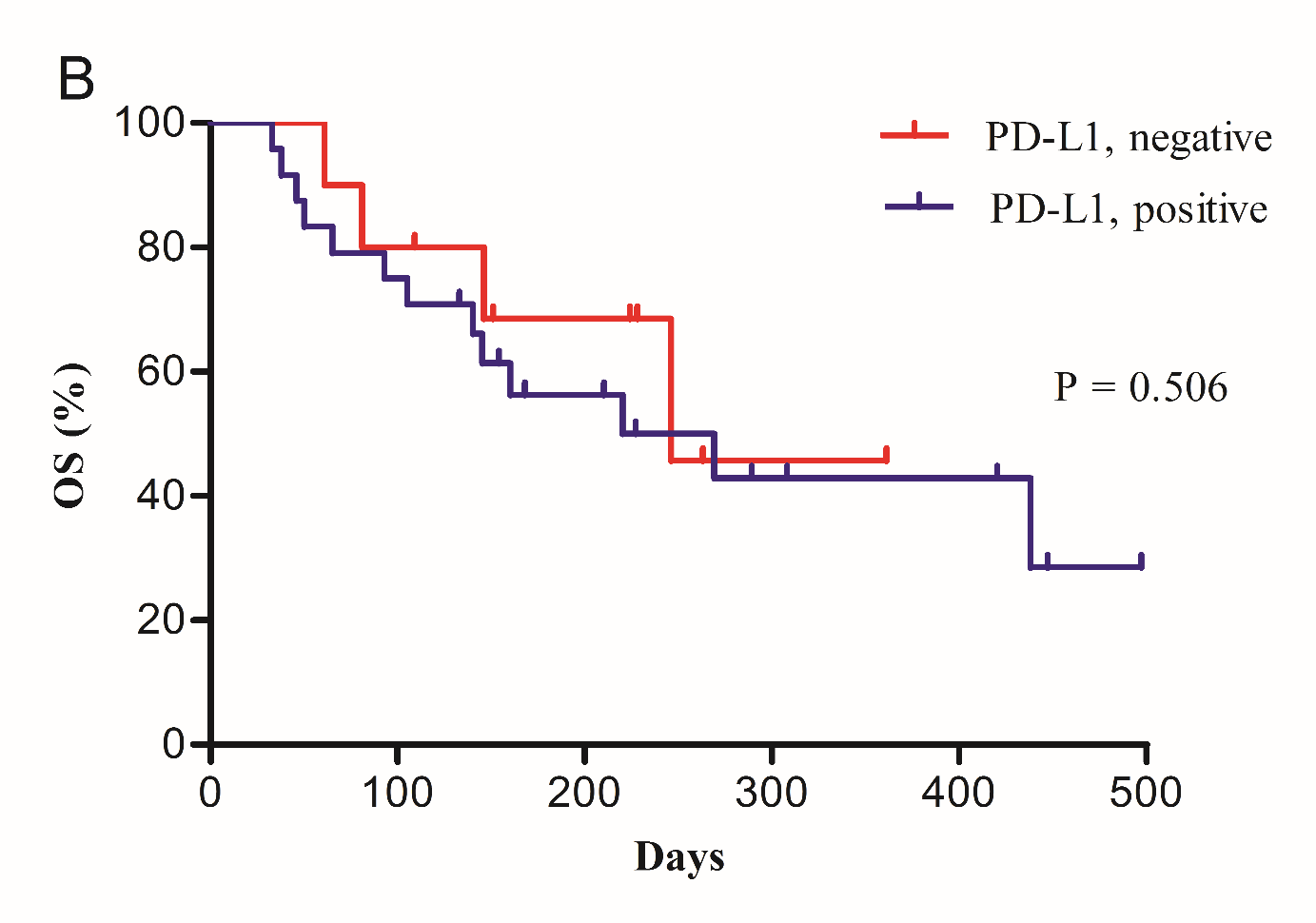


**Supplementary Table 1.** Progression-free and overall survival according to PD-L1 expression.

|  |  | **PD-L1+ (n = 24)** | **PD-L1− (n = 10)** | **Total (n = 34)** | **P-value*** |  |
| --- | --- | --- | --- | --- | --- | --- |
|  | **Progression-free survival (PFS)** |  |  |  | 0.423 |  |
|  | Patients with event, n (%) | 18 (75) | 9 (90) | 27 (79.4) |  |  |
|  | Median PFS, days (IQR) | 66 (37, 146) | 49 (40, 81) | 65 (37, 146) |  |  |
|  | Mean PFS, days (95% CI) | 167 (89, 244) | 78 (41, 115) | 146 (85, 206) |  |  |
|  | **Overall survival (OS)** |  |  |  | 0.631 |  |
|  | Patients with event, n (%) | 13 (54.2) | 4 (40) | 17 (50) |  |  |
|  | Median OS, days (IQR) | 269 (93, N/E) | 246 (146, N/E) | 246 (105, N/E) |  |  |
|  | Mean OS, days (95% CI) | 277 (198, 357) | 252 (173, 331) | 285 (218, 353) |  |  |

* Log-rank test
